# The Financial Burden of Non-Communicable Chronic Diseases in Rural Nigeria: Wealth and Gender Heterogeneity in Health Care Utilization and Health Expenditures

**DOI:** 10.1371/journal.pone.0166121

**Published:** 2016-11-10

**Authors:** Wendy Janssens, Jann Goedecke, Godelieve J. de Bree, Sunday A. Aderibigbe, Tanimola M. Akande, Alice Mesnard

**Affiliations:** 1 Faculty of Economics, VU University, Amsterdam, The Netherlands; 2 Tinbergen Institute, Amsterdam, The Netherlands; 3 Amsterdam Institute for International Development, Amsterdam, The Netherlands; 4 Faculty of Economics and Business, KU Leuven, Campus Brussel, Brussels, Belgium; 5 School of Business and Law, University of Agder, Kristiansand, Norway; 6 PharmAccess Foundation, Amsterdam, The Netherlands; 7 Center for Tropical and Travel Medicine, Academic Medical Center, University of Amsterdam, Amsterdam, The Netherlands; 8 Department of Epidemiology and Community Health, University of Ilorin Teaching Hospital, Ilorin, Nigeria; 9 City University London, London, United Kingdom; 10 Institute for Fiscal Studies, London, United Kingdom; 11 CEPR and Center for Global Development, London, United Kingdom; RTI International, UNITED STATES

## Abstract

**Objectives:**

Better insights into health care utilization and out-of-pocket expenditures for non-communicable chronic diseases (NCCD) are needed to develop accessible health care and limit the increasing financial burden of NCCDs in Sub-Saharan Africa.

**Methods:**

A household survey was conducted in rural Kwara State, Nigeria, among 5,761 individuals. Data were obtained using biomedical and socio-economic questionnaires. Health care utilization, NCCD-related health expenditures and distances to health care providers were compared by sex and by wealth quintile, and a Heckman regression model was used to estimate health expenditures taking selection bias in health care utilization into account.

**Results:**

The prevalence of NCCDs in our sample was 6.2%. NCCD-affected individuals from the wealthiest quintile utilized formal health care nearly twice as often as those from the lowest quintile (87.8% vs 46.2%, p = 0.002). Women reported foregone formal care more often than men (43.5% vs. 27.0%, p = 0.058). Health expenditures relative to annual consumption of the poorest quintile exceeded those of the highest quintile 2.2-fold, and the poorest quintile exhibited a higher rate of catastrophic health spending (10.8% among NCCD-affected households) than the three upper quintiles (4.2% to 6.7%). Long travel distances to the nearest provider, highest for the poorest quintile, were a significant deterrent to seeking care. Using distance to the nearest facility as instrument to account for selection into health care utilization, we estimated out-of-pocket health care expenditures for NCCDs to be significantly higher in the lowest wealth quintile compared to the three upper quintiles.

**Conclusions:**

Facing potentially high health care costs and poor accessibility of health care facilities, many individuals suffering from NCCDs—particularly women and the poor—forego formal care, thereby increasing the risk of more severe illness in the future. When seeking care, the poor spend less on treatment than the rich, suggestive of lower quality care, while their expenditures represent a higher share of their annual household consumption. This calls for targeted interventions that enhance health care accessibility and provide financial protection from the consequences of NCCDs, especially for vulnerable populations.

## Introduction

Non-communicable chronic diseases (NCCDs) pose an increasing burden on low- and middle-income countries (LMICs) [1]. The World Health Organization estimates that 60% of all deaths in the world can be attributed to chronic disease and that 70% of these fatalities occur in LMICs [2]. Both men and women in LMICs are heavily affected, with death rates related to chronic disease estimated to be 54% and 86% higher, respectively, compared to men and women in developed countries [3]. It is estimated that the increasing prevalence of NCCDs will have substantial macroeconomic consequences for LMICs in the coming decades [3]. However, much less is known about the financial burden of NCCDs at the micro-level, including for Africa [4].

Despite the high prevalence of NCCDs in LMICs, the majority of the population lacks access to affordable good quality health services for prevention, treatment and care [5]. Financial and geographic factors are major determinants of limited accessibility to health care in general but related evidence on NCCDs is more limited.

High cost of treatment of NCCDs may represent a considerable barrier to care. A study conducted in India found that among rural households, costs incurred due to non-communicable diseases represent a higher income share than those incurred due to communicable diseases [6]. In a study of 35 low-income countries, individuals with diabetes were found to be at high risk of catastrophic health spending [7]. Likewise, studies in Bangladesh [8], Burkina Faso [9], and Georgia [10] found that the risk of financial catastrophe increased significantly for households with a chronically ill member.

Most of these studies, however, do not investigate interrelations of health care expenditures, utilization and accessibility, and they typically do not disaggregate expenditures and treatment seeking behavior for NCCDs by wealth indicators. Whereas the poor are as likely as the non-poor in LMICs to suffer from NCCDs [11], their financial capacity to cope with chronic diseases is by definition more constrained. In the context of hospitalization for cardiovascular diseases, it was shown that poorer households in four LMICs had significantly higher expenditures in terms of costs relative to income compared to richer households [12]. [10] show that the incidence of catastrophic health spending among chronically ill households is largest in the poorest quintile. Taken together, these studies suggest that the financial strain caused by NCCDs put especially the poorest households at risk.

Due to their long-term nature, NCCDs generally require recurrent visits to the health care provider, underscoring the importance of geographical accessibility. The evidence on expenditures for acute illness shows that transportation costs are a non-negligible component of total health expenditures [13]. Long distance trips to health care providers have been shown to impede poor households from seeking care in a wide variety of countries, including Nigeria [14], Eritrea [15], India [16,17] and Côte d’Ivoire [18]. The possession of a motorized vehicle has been shown to increase the likelihood of seeking health care [19]. This emphasizes the link between access to care and mobility mediated by income.

Overall, these studies indicate that both the direct medical costs of treatment and indirect costs such as travel expenses limit the affordability of health care for many people in LMICs. Chronic diseases induce recurrent and long-term costs, exacerbating financial pressures on disadvantaged households. We hypothesize that households at highest risk of financial problems are those in settings that both have a low density of good quality clinics and a lack of financial protection mechanisms against health shocks such as social, community-based or private health insurance.

The present study aims to provide insights into the interrelation of NCCD-related health care expenditures, health care seeking behavior and health care accessibility. Specifically, a Heckman selection framework is employed to estimate health care expenditures after sample-selection bias has been corrected for. Further, the study assesses whether poor compared to wealthier individuals and women compared to men are affected differentially. It analyses out-of-pocket expenditures for treatment and for transportation separately, as well as the incidence of catastrophic health spending due to NCCDs.

The study focuses on a rural part of western Nigeria. Nigeria is still characterized by a high poverty rate particularly among the rural population, a weak health care system, and long travel distances to health facilities in vast rural areas. Non-communicable chronic diseases are estimated to account for 24% of total deaths in the country [20]. Elderly households, which are typically more exposed to chronic illness, are particularly at risk to incur catastrophic health expenditures [21]. Nigerian women may suffer disproportionately from a lack of access to affordable and good quality health care. Maternal mortality rates in Nigeria are among the highest in the world [22]. To date, little is known about the vulnerability of the poor and of women in Nigeria to the financial consequences of chronic disease.

## Methods

### Study design, study population and data collection

The data in this study were collected as part of a cross-sectional household survey conducted in 2009 among a largely rural population in central Kwara State, Nigeria. The household survey was conducted as a baseline measurement for the subsequent implementation of a low-cost health insurance program of the Health Insurance Fund [23]. The survey was set up as a random population-based sample of Afon and Ajasse district.

The target sample size was 1500 households, stratified into two groups of equal sample size. The first stratum consisted of households living in communities with a potential insurance program clinic within their boundaries. The second group were households residing outside such communities but with a maximum distance of 15 kilometers to the nearest potential clinic. A two-staged self-weighted sample was drawn. After 100 enumeration areas were randomly selected from the 2006 National Population Census with probability proportional to size, an average of 15 households was randomly sampled per enumeration area, the exact number depending on the size of the enumeration area to represent the population density in the sample.

The dataset includes three sections: 1) information on demographics, education, food and non-food consumption as well as global position system (GPS) codes of household residences; 2) bio-medical information on health status, health care utilization, and health-related expenditures; and 3) information from a health facility census including GPS codes of the 110 facilities attended by the respondents in the twelve months preceding the survey.

### Definition of the main outcome variables

#### Non-communicable chronic diseases

Chronic diseases can be classified as either non-communicable, such as cardiovascular disease or diabetes, or as communicable, such as HIV/AIDS. This article focuses on the former. Biomedical interviews were conducted with all household members to obtain self-reported health status regarding chronic diseases. Respondents were asked if they currently suffered from any of the following chronic diseases, with clarifications given by interviewers as specified in parentheses: cardiovascular disease (heart disease, having had a myocardial attack or heart failure), hypertension (high blood pressure), chronic respiratory disease (asthma, disease of the lungs from early years or since childhood with episodes of breathlessness), diabetes (sugar disease or sugar sickness), musculoskeletal disorders (including low back pain, arthritis, disease of the joints with swelling and pain, spinal disorders, and trauma), epilepsy (having regular seizures), allergy, sickle cell disease (disease of red blood cells with episodes of anemia and pain in parts of the body), physical disability, or any other chronic disease. Responses in the “other” category are only included to the extent that they represent non-communicable chronic diseases.

The most frequently reported “other” chronic disease was peptic ulcer disease and is therefore documented as a separate category. Epilepsy and sickle cell disease occurred rarely and were therefore merged with “other” chronic diseases. Further, hypertension was counted among cardiovascular diseases.

#### Asset-based wealth quintile

A proxy for socio-economic status was constructed using an asset-based wealth index to capture longer-term wealth [24], as has been used before in studies on health care in low-income countries [25,26]. The wealth index is based on the first factor loading of a principal component analysis of a large set of asset variables and housing characteristics, including details on the housing situation (such as home ownership status, quality of walls, number of rooms, sanitation), durable assets (such as numbers of TVs, fridges, paraffin lamps, bicycles), machinery (irrigation equipment, ploughs, tractors etc.) and livestock. Individuals were ranked according to their wealth index value and divided into quintiles with the poorest individuals in the first quintile. The Pearson correlation between the wealth index and log annual household consumption was .57, and the Spearman correlation of wealth and education in ordinal scale was .31.

#### Health care utilization

Information was collected on health care utilization, provider choice, medical expenditures, and travel costs in the twelve months prior to the survey. Formal health care was defined as being supplied by a public or private health provider. Public health providers included primary health centers (including maternal and child health clinics), secondary facilities, and tertiary public hospitals. Private health providers included private doctors, nurses, and midwives, as well as pharmacies, and private and missionary clinics. Informal health care utilization comprised the purchase of medicines from unqualified patent medicine vendors or drugs stalls, or visiting a traditional healer. The residual category was not seeking care, either formally or informally.

#### Health care expenditures

Health care expenditures were split in two categories: out-of-pocket health expenditures (OOPs) and travel costs. OOPs encompass expenses for consultations, treatment, medicines, cost of stay, laboratory and diagnostic tests, and other direct costs. They include costs paid by others (e.g. through gifts) but exclude costs covered by a health insurance scheme. Since the survey was conducted before the health insurance scheme was introduced in one of the sampled districts, insurance coverage was very low (below 1%). Travel costs capture expenditures for transportation to and from the health care provider and include transportation costs for accompanying persons. Expenditures for chronic diseases were calculated as total annual expenses for the NCCD that most affected the respondent’s daily life.

Relative expenditures were defined as total annual expenditures for the treatment of chronic disease divided by total annual household consumption (AHC). Consumption included food consumption (either purchased, free, or self-produced) and a comprehensive list of non-food items (either purchased or received for free), including spending on health. For goods either received for free or self-produced, respondents were asked to report Naira equivalents. Amounts that were captured in weekly or monthly terms were annualized.

#### Catastrophic health care spending

NCCD-related catastrophic health care spending (CHS) was assessed at the household level, therefore expenditures of all NCCD-affected household members were summed. Health expenditures are understood as catastrophic if they represent a significant share of a household’s total disposable income. There is no general consensus about which share this would be and about the underlying measure of income, and existing measures continue to be challenged [27–29]. In this study we follow the definition of [30], who define out-of-pocket health costs to be catastrophic if they exceed 40% of the households’ capacity to pay. The capacity to pay reflects the total household income, typically proxied by consumption, after subsistence needs have been met.

Following [30], we estimated the general level of subsistence needs for any household as the average spending on food for those households whose share of food expenditures in their total consumption was in the 45^th^ to 55^th^ percentile. This ensures that the estimate for subsistence expenditures is not driven by a single household (such as the median). The resulting estimate for subsistence needs was adjusted for each household according to its size using an estimated consumption equivalence scale. This takes into account that food consumption typically increases less than proportional with every additional household member. The specification is similar to the one used in [30] except for their country-level fixed effects, since we do not use international data:

log(HH food consumption)=log α+β log(HHsize)+ϵ

We estimated the elasticity of household consumption with respect to additional household members, *β*, to be .42 (SE = .021; p<0.001), which is of a similar order of magnitude as in [30], whose point estimate of the elasticity is .56.

#### Distances to health care facilities

**Fig 1:**
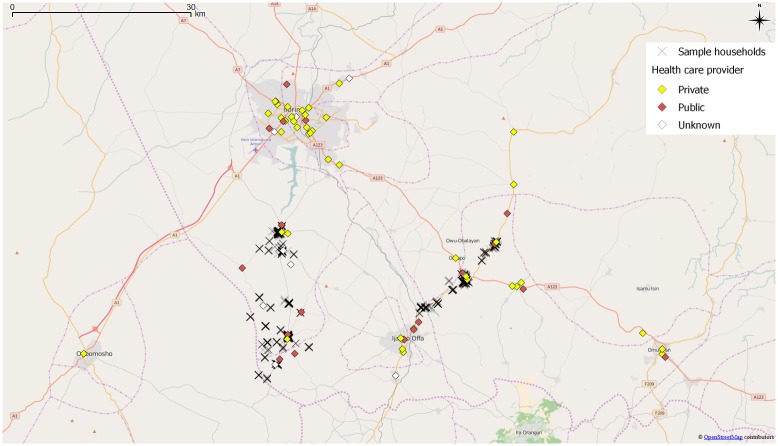
Geographical distribution of surveyed households and health care facilities. Source: Openstreetmap.org, authors’ survey.

Travel distances to the nearest health provider and to the health provider of choice were measured using the GPS coordinates of household dwellings and health care providers. Employing the engine from Open Source Routing Machine (OSRM, see http://project-osrm.org/, accessed on September 22, 2016), we drew on the road network data provided by OpenStreetMap (obtained from http://download.geofabrik.de/africa.html, accessed on the same date), to calculate the closest overland routes between dwellings and facilities [31]. OpenStreetMap data has been extensively used in research related to geographic information science, as discussed by [32]. Fig 1 shows the spatial distribution of respondents and health care facilities included in the study.

### Data analysis

Data were analyzed using Stata version 13.1. The unit of observation was the individual respondent. All standard errors were clustered at the level of the enumeration areas. P-values below 0.05 were considered statistically significant and 95% confidence intervals are shown. For proportion estimates, we calculated cluster-adjusted Agresti-Coull confidence intervals, which account for non-normality in the distribution of proportions [33] and which have been found to outperform standard Wald confidence intervals in coverage probability [34]. Mean comparisons were carried out by using two-tailed Wald tests; categorical distributions were compared using χ^2^-tests.

The analyses of absolute and relative expenditures are conducted unconditional on seeking care, i.e. they include the full sample of chronically ill individuals independent of their health-seeking behavior. Similar to [8], we take into account that we observe (zero or non-zero) health care expenditures only for the subset of chronically ill respondents who visited a health care provider. Whereas [8] treat health care seeking behavior as a double hurdle decision process, we go one step further and use a Heckman selection model to explicitly model whether an ill individual seeks care and thus “participates” in the equation that determines out-of-pocket health care costs. In the main regression (1), we estimate how expenditures vary by sex, wealth quintile and additional household characteristics for individuals who sought formal care. This information is only observed for those individuals who actually seek care. In the Selection (“Participation”) Eq (2), we model whether formal health care is sought using distance to the nearest formal health facility as Heckman instrument, denoted by *Z*_*j*_ in Eq (2). *S*_*j*_ is a dummy variable indicating “participation” of observation *j* in the main regression. The random error terms of both equations are governed by the joint normal distribution (3), which links the main regression and the selection equation by allowing for a nonzero correlation *ρ* of their respective error terms.

$$OOP_{j}=X_{j}\beta +\epsilon_{j},\;OOP_{j}\;observed\;if\;S_{j}=1$$ (1)

$$S_{j}=1(\;Z_{j}\gamma +X_{j}\delta +\nu_{j}>0)$$ (2)

$$\left(\epsilon_{j},\;\nu_{j}\right)\sim N\left(0,\Sigma \right)\;with\;\Sigma =\;\left(\begin{array}{cc} \sigma^{2} & \rho \sigma \\ \rho \sigma & 1 \end{array}\right)$$ (3)

Coefficients were estimated simultaneously through maximization of the full information likelihood function, which yields more efficient estimates than the traditional two-step case [35]. We performed Wald χ^2^ tests to test whether the null of *ρ* = 0 could be rejected, which is tantamount to testing for independence of the unobservable error terms of (1) and (2). This Heckman selection model allows us to control for the selection of the sample that may otherwise bias the estimates when independence does not hold.

Since transportation costs are directly correlated with the distance to be travelled, hence violating the exclusion restriction for the instrumental variable (see the discussion below), travel expenditures are not analyzed with a Heckman selection model.

Given that expenditures are right-skewed and that we observe a considerable share of reported zero expenditures, we use their inverse hyperbolic sine (IHS) transform in the regressions [36]. The advantage of an IHS transformation over a standard logarithmic transformation is that the former is defined at zero in contrast to the latter, while regression estimates can be interpreted in the same way for both. The IHS transform z* of a variable *z* is given by:

$$z^{*}=\text{log}\left(z+\sqrt{z^{2}+1}\right)$$

### Ethical clearance

The Kwara State Ministry of Health and the Ethical Review Committee of the University of Ilorin Teaching Hospital approved the study. All adult participants either signed or thumb-printed a written informed consent form. Information from children up to 18 years old was collected after written consent from their parents, either by direct interview (with adolescents aged 12–18 years) or by interview with their parents.

## Results

### Population characteristics

The interviewed sample consisted of 1,450 households, 884 in Afon district and 566 in Ajasse district. Fifty households were either not found, inhabitants were absent, or they refused to participate. Forty-three households were deleted from the dataset because of data quality concerns due to technical problems in the field. The final sample hence includes 1,407 households, encompassing 5,761 individuals. This corresponds to an average household size of 4.1. Additional population characteristics are shown in Table 1 column 1.

**Table 1:** Demographic, socio-economic and health characteristics of the population.

	Total sample	Individuals suffering from chronic diseases		
		%	[95% CI] ^§^	N
**Total**	5761	6.2%	[5.3%; 7.2%]	356
**Sex**				
Male	2763	5.4%	[4.4%; 6.7%]	150
Female	2983	6.9%	[5.9%; 8.1%]	206
**Age Category**				
0–9	1708	0.4%	[0.1%; 0.9%]	6
10–19	1335	1.6%	[1.0%; 2.7%]	22
20–29	526	3.8%	[2.4%; 5.8%]	20
30–39	495	5.5%	[3.6%; 8.2%]	27
40–49	522	10.3%	[7.5%; 14.1%]	54
50–59	416	15.9%	[12.2%; 20.4%]	66
60–69	380	16.3%	[12.3%; 21.4%]	62
70+	367	27.0%	[22.1%; 32.5%]	99
**Highest Completed Education**				
No Education	2528	9.0%	[7.7%; 10.6%]	228
Primary incomplete	1160	1.8%	[1.2%; 2.8%]	21
Primary complete	614	6.4%	[4.3%; 9.3%]	39
Secondary incomplete	703	2.8%	[1.7%; 4.7%]	20
Secondary complete	440	5.9%	[3.9%; 8.8%]	26
Tertiary	262	7.3%	[4.5%; 11.3%]	19
Other	38	5.3%	[0.5%; 18.2%]	2
**Head of household**				
Male	4751	5.7%	[4.8%; 6.7%]	269
Female	1010	8.6%	[6.8%; 10.9%]	87
**Asset-based wealth quintile** (average annual household consumption in Naira given in parentheses)				
1 (₦ 215,565)	1154	8.3%	[6.8%; 10.2%]	96
2 (₦ 301,986)	1151	6.6%	[5.0%; 8.7%]	76
3 (₦ 363,311)	1152	6.9%	[5.0%; 9.3%]	79
4 (₦ 475,647)	1154	4.7%	[3.4%; 6.5%]	54
5 (₦ 669,638)	1149	4.4%	[3.1%; 6.3%]	51

^§^ Agresti-Coull confidence intervals are adjusted for clustering at the enumeration area level.

The exchange rate on July 1, 2009, was USD 0.68 for 100 Naira. When converting annual household consumption into USD equivalents and dividing by household size, we obtain approximate poverty levels comparable to national levels in Nigeria: 51.5% of the sample population lived below USD 1.25 per day versus 62% according to the country-wide estimate, and 77.2% of the sample lived below USD 2 per day compared to 82.2% in the entire country [37]. Less than 1% of the respondents were insured against health expenditures at the time of the survey, slightly less than the 1.8% insurance coverage country-wide [38].

Wealth inequality in the sample population captured by the asset-based quintiles was mirrored in large differences in consumption across the quintiles. Total annual household consumption (not adjusted for household size) for the poorest quintile was estimated to be the equivalent of 215,565 Naira (USD 1,465) while for the richest quintile the figure amounted to 669,638 Naira (USD 4,553), thus more than three times as much as for the poorest quintile.

### The prevalence of chronic disease

Table 1 column 2 shows the self-reported prevalence of chronic diseases at the time of the survey. Overall, 6.2% of the study population reported suffering from at least one chronic condition. Musculoskeletal disorders were most prevalent (45.8%), followed by cardiovascular diseases (13.7%) (see S1 Table). Women were more likely than men to report an NCCD (6.9% and 5.4%, respectively), although the difference is not statistically significant. Prevalence increased with age, with 27.0% of individuals aged 70 or above reporting a chronic disease. Individuals without any education reported an NCCD more often than educated individuals, but no clear pattern in prevalence arose between more disaggregated levels of education. The prevalence of NCCD was highest in the poorest wealth quintile (8.3%) and lowest in the richest quintile (4.4%).

### Health care utilization

On average, 63.3% of individuals reporting an NCCD visited a formal health care provider at least once in the year prior to the survey (Table 2). Males were more likely to seek formal care than females (73.0% vs. 56.5%), yet due to the relatively small overall number the 5% significance threshold is not surpassed. But one third of all women in the sample suffering from an NCCD sought informal care, compared to only roughly a fifth of men. Although men and women reported distributions over types of NCCDs that were different in a statistical sense (χ^2^ [df = 7]: 14.13, p = 0.049), distributional patterns were fairly comparable in relative terms (see Appendix B), such that the observed difference in health care utilization cannot be driven purely by heterogeneity in disease patterns.

**Table 2:** Healthcare utilization for individuals with an NCCD.

	NCCD-affected individuals	Total who seek care				*Among those who seek formal care*	
		Informal		Formal		*Public*	*Private or missionary*
	N	Pct	[95% CI] ^§^	Pct	[95% CI] ^§^	Pct	Pct
**Total**	341	27.6	[21.6; 34.5]	63.3	[56.2; 69.9]	43.1	56.9
**Sex**							
Male	141	19.1	[12.6; 27.9]	73.0	[63.3; 81.0]	43.7	56.3
Female	200	33.5	[25.5; 42.6]	56.5	[47.7; 64.9]	42.5	57.5
**Asset-based wealth Quintile**							
1 (poorest)	93	43.0	[32.6; 54.1]	46.2	[36.2; 56.6]	37.2	62.8
2	74	37.8	[26.9; 50.1]	47.3	[35.4; 59.5]	40.0	60.0
3	75	24.0	[13.7; 38.5]	72.0	[57.0; 83.4]	38.9	61.1
4	50	14.0	[5.2; 30.8]	82.0	[65.2; 92.0]	53.7	46.3
5 (richest)	49	2.0	[0.0; 12.0]	87.8	[73.6; 95.2]	46.5	53.5

^§^ Agresti-Coull confidence intervals are adjusted for clustering at the enumeration area level. Residual category (not displayed) is ‘not seeking care’.

Formal health care utilization was monotonically increasing across wealth quintiles, with 46.2% of poorest individuals seeking formal care compared to 87.8% of richest individuals (significant at the 5% error level). The opposite picture was drawn by utilization of informal care: 43% of NCCD-affected households in the poorest wealth quintile sought informal care, contrasting with mere 2% of those in the upper quintile.

Among those who visited a formal health care provider, private facilities were consulted more frequently than public facilities, at 56.9% and 43.1%, respectively (Table 2, columns 4–7). Both men and women consulted private and public providers to the same degree. In contrast, individuals in the poorest wealth quintile were more likely to visit private instead of public providers compared to the richest quintile.

### Out-of-pocket expenditures (OOPs), transportation costs and catastrophic health care spending (CHS)

To evaluate the financial burden of health care, the amount of treatment-related OOPs and transportation costs were calculated both in absolute terms (in Naira) and relative to annual household consumption (AHC) (Table 3). Individuals who suffered from an NCCD spent on average 9,889 Naira on treatment, or 4.04% of AHC. This amount can be broken down into 9,117 Naira (3.75% of AHC) spent on medical out-of-pocket expenditures (OOPs) and 819 Naira (0.31% of AHC) spent on transportation. Differences between male and female expenditures were not statistically significant (p = 0.822 and p = 0.762, for absolute and relative total expenditures, respectively).

**Table 3:** Absolute and relative expenditures for NCCDs.

*Panel A*						
	Absolute costs of seeking healthcare for NCCD, in Nigerian Naira					
	OOP	*Std*. *err*.	Transport	*Std*. *err*.	Total	*Std*. *err*.
**Total**	9,117	(1,173)	819	(111)	9,889	(1,232)
**Sex**						
Male	8,930	(1,398)	701	(142)	9,611	(1,461)
Female	9,248	(1,616)	901	(156)	10,084	(1,704)
**Asset-based wealth quintile**						
1 (poorest)	8,866	(2,182)	553	(132)	9,407	(2,229)
2	7,006	(1,640)	771	(201)	7,766	(1,756)
3	9,928	(2,000)	950	(269)	10,853	(2,234)
4	6,441	(1,620)	912	(285)	7,181	(1,694)
5 (richest)	14,227	(4,922)	1,120	(285)	15,323	(5,047)
Observations	334		327		335	
*Panel B*						
	Relative costs as % of total annual household consumption					
	OOP	*Std*. *err*.	Transport	*Std*. *err*.	Total	*Std*. *err*.
**Total**	3.75	*(0*.*57)*	0.31	*(0*.*04)*	4.04	*(0*.*59)*
**Sex**						
Male	3.58	*(0*.*69)*	0.26	*(0*.*06)*	3.84	*(0*.*72)*
Female	3.87	*(0*.*86)*	0.35	*(0*.*06)*	4.19	*(0*.*89)*
**Asset-based wealth quintile**						
1 (poorest)	5.86	*(1*.*80)*	0.31	*(0*.*07)*	6.16	*(1*.*83)*
2	3.01	*(0*.*79)*	0.31	*(0*.*09)*	3.32	*(0*.*84)*
3	3.87	*(0*.*96)*	0.39	*(0*.*12)*	4.24	*(1*.*07)*
4	1.78	*(0*.*44)*	0.23	*(0*.*06)*	1.97	*(0*.*46)*
5 (richest)	2.50	*(0*.*71)*	0.27	*(0*.*09)*	2.76	*(0*.*77)*
Observations	334		327		335	

*Notes*: Wald standard errors adjusted for clustering at the enumeration area level.

Expenditure data are missing for some observations; outliers above 99^th^ percentile are excluded.

**Fig 2:**
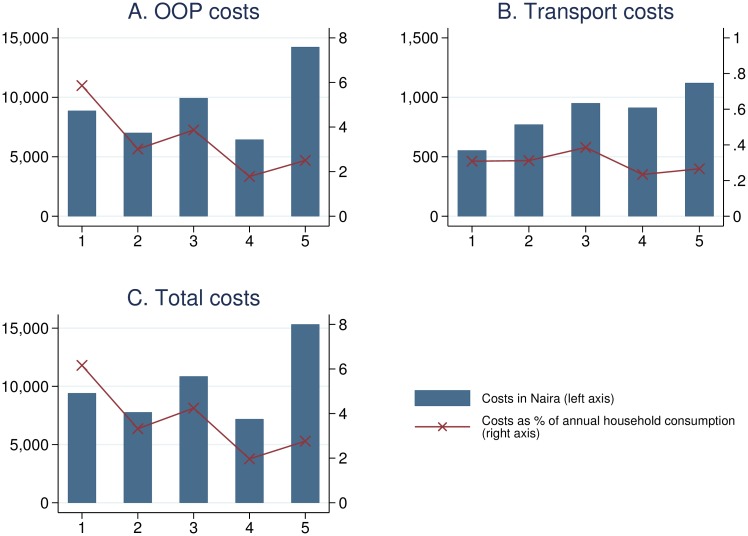
Health expenditures for NCCDs by wealth quintile. Costs trimmed at 99^th^ percentile. The blue bars show average expenditures in Nigerian Naira for each asset-based wealth quintile, in total as well as broken down into out-of-pocket and transport costs. The exchange rate at the time of the survey was approximately USD 0.68 for 100 Naira. The red lines indicate the expenditures as percentage of total annual household consumption.

Fig 2 Panel A shows OOPs by wealth quintile. The poorest quintile paid more out of pocket than the second and fourth quintile, but less than the third quintile, while the richest paid most in absolute terms, reflecting its higher capacity to pay for it. However, in relative terms, the lowest quintile incurs 5.86% of total household annual consumption, which is more than any other quintile. Transport costs, depicted in Fig 2 Panel B, display an increasing trend among wealth quintiles, which corresponds to the lower rate of formal health care utilization for lower quintiles. Overall, as shown in Fig 2 Panel C, the lowest quintile pays the largest share of annual household consumption (6.16%) and more than twice the share of the richest quintile (2.76%). The numbers corresponding to Fig 2 are summarized in Table 3.

The incidence of catastrophically high health care spending for treatment of NCCDs, defined as exceeding 40% of a household’s annual capacity to pay, is depicted in Table 4. Women were more vulnerable to suffer from catastrophic health spending (CHS) at 9.5% compared to men at 7.9%, but not statistically significantly. Households in the lower two wealth quintiles were more likely to incur CHS (at 10.8% and 13.5%, respectively) than richer households.

**Table 4:** Catastrophic health spending (CHS) on NCCDs.

	N	Proportion of CHS (in pct)	[95% CI]^§^
Total	339	8.8	[5.7; 13.4]
**Sex**			
Male	140	7.9	[4.0; 14.4]
Female	199	9.5	[5.9; 15.0]
**Asset-based wealth quintile**			
1 (poorest)	93	10.8	[5.7; 18.9]
2	74	13.5	[6.0; 26.7]
3	75	6.7	[2.2; 16.2]
4	49	6.1	[0.4; 22.5]
5 (richest)	48	4.2	[0.0; 22.1]

^§^ Agresti-Coull confidence intervals are adjusted for clustering at the enumeration area level. Health spending on NCCDs is defined as ‘catastrophic’ if it exceeds 40% of the household’s estimated annual capacity to pay.

### Travel distance to health care facilities

The average distance to the nearest facility of any type was 1.8 kilometers (Table 5 column 1). The distances to both the closest private and public facilities were 2.5 kilometers on average (Table 5 columns 3–5), such that both types were equally accessible in geographical terms. The distances monotonically declined with increasing wealth quintiles, with the poorest quintile living furthest away (2.6 kilometers, column 1) and the richest quintile living closest to a facility (1.0 kilometers; Wald: 29.5, p<0.001). No difference was found between sexes. The negative correlation between wealth and distance was confirmed when looking at public and private providers separately.

**Table 5:** Mean distances to nearest facilities and distances actually travelled to seek treatment for chronic disease.

	Distance to nearest facility						Distance travelled (in km)	
	Total	*Std*. *err*.	public	*Std*. *err*.	private	*Std*. *err*.	Total	*Std*. *err*.
**Total**	1.76	*(0*.*20)*	2.45	*(0*.*26)*	2.47	*(0*.*21)*	14.10	*(1*.*50)*
**Sex**								
Male	1.79	*(0*.*22)*	2.52	*(0*.*28)*	2.50	*(0*.*22)*	13.83	*(1*.*75)*
Female	1.73	*(0*.*19)*	2.38	*(0*.*24)*	2.45	*(0*.*20)*	14.39	*(2*.*23)*
**Asset-based wealth quintile**								
1 (poorest)	2.55	*(0*.*30)*	3.83	*(0*.*39)*	3.57	*(0*.*28)*	12.62	*(2*.*99)*
2	2.51	*(0*.*29)*	3.28	*(0*.*32)*	3.15	*(0*.*27)*	13.06	*(3*.*32)*
3	1.56	*(0*.*20)*	2.31	*(0*.*31)*	2.31	*(0*.*23)*	13.45	*(2*.*78)*
4	1.18	*(0*.*15)*	1.54	*(0*.*19)*	1.79	*(0*.*17)*	16.58	*(3*.*16)*
5 (richest)	1.00	*(0*.*12)*	1.28	*(0*.*16)*	1.54	*(0*.*17)*	14.38	*(3*.*76)*
Observations	5758		5758		5758		179	

*Notes*: Unit is kilometres. Wald standard errors are adjusted for clustering at the level of an enumeration area.

The distances actually travelled to the chosen provider were much larger than the distances to the nearest facility (Table 5 columns 7–8), averaging 14.1 kilometers. Women travelled somewhat further on average (14.4 kilometers, compared to 13.8 kilometers for men), but not significantly. The lowest wealth quintile covered the shortest distance (12.6 kilometers), despite living furthest away from the nearest facility, while individuals in the highest quintile travelled 1.8 kilometers more on average. Within-group variation in actually travelled distances was large however, such that differences were not statistically significant. It is interesting to note that overall, only 10% of the chronically ill opted for their nearest facility.

### The relation between OOP expenditures, health care utilization and distance to facilities

The Heckman selection model combines health expenditures, formal health care utilization and geographical accessibility in one model. Table 6 shows the results of the Heckman model to estimate determinants of health expenditures, using distance to the nearest health care provider as instrumental variable to explain formal health care utilization. The odd columns show the estimates of the main regression Eq (1), the even columns show the estimates of the selection equation Eq (2). The dependent variable in the first model (columns 1 and 2) is the absolute OOPs for health care, including wealth quintiles and individual as well as household characteristics as regressors. The dependent variable in the second model (columns 3 and 4) is OOPs for health care as percent of annual household consumption.

**Table 6:** Heckman selection model for out-of-pocket (OOP) healthcare expenditures.

	OOP		OOP as pct of annual consumption	
	*Main model*	*Selection equation*	*Main model*	*Selection equation*
Distance to nearest facility		-0.0556*		-0.0910**
		(0.0285)		(0.0409)
Female	1.266**	-0.520***	0.0606	-0.611***
	(0.519)	(0.159)	(0.258)	(0.182)
Wealth quintile (‘poorest’ is ref. cat.)				
2	0.845	0.00822	0.384	0.0553
	(0.611)	(0.217)	(0.280)	(0.245)
3	-1.424**	0.833***	0.0633	0.740**
	(0.653)	(0.249)	(0.328)	(0.296)
4	-1.164*	0.829***	-0.0460	0.919***
	(0.598)	(0.271)	(0.332)	(0.325)
5	-1.808**	1.098***	-0.309	1.236***
	(0.714)	(0.265)	(0.365)	(0.351)
Household size	0.126	-0.0250	0.00960	-0.0298
	(0.0890)	(0.0312)	(0.0352)	(0.0362)
Age	0.0350***	-0.00218	0.0179***	-0.00226
	(0.0112)	(0.00420)	(0.00470)	(0.00455)
Female Head of HH	-0.512	0.407**	0.345	0.551***
	(0.580)	(0.183)	(0.289)	(0.209)
Education (‘none’ is ref. cat.)				
Primary	-0.238	0.266	0.171	0.193
	(0.512)	(0.197)	(0.223)	(0.218)
Secondary or higher	0.646	0.361	0.362	0.161
	(0.507)	(0.249)	(0.268)	(0.306)
Observations	339		339	
*ρ*	-0.948		-0.085	
Wald χ**²** independent equations test (df)	55.23***	(1)	0.170	(1)
*p*-value	0.000		0.680	
Wald χ² model fit (df)	22.43**	(10)	28.12***	(10)
*p*-value	.013		.002	

*Notes*: Standard errors adjusted for clustering at the enumeration area level are given in parentheses. *p*-values: * 10%, ** 5% and *** 1%. Estimates obtained by maximizing the full information likelihood function. *ρ* is the correlation of the error terms of main regression and selection equation. ‘df’ are degrees of freedom.

As expected, the probability of seeking formal care depends significantly and negatively on the distance to the nearest facility in both Participation equations (Table 6 columns 2 and 4), which confirms that our identifying instrument is powerful. Further, women are significantly less likely to seek formal care, while households from the upper three wealth quintiles as well as individuals from female-headed households are significantly more likely.

We find strong evidence of a selection effect in the first model explaining absolute OOPs (Table 6, columns 1 and 2). This is shown by the correlation between the error terms, which is high and negative (*ρ* = –.95, p<0.001). In the main regression of absolute OOPs (Table 6 column 1), we observe significantly negative coefficients for the three upper wealth quintiles compared to the poorest quintile. This implies that, after accounting for self-selection into treatment, the absolute expenditures for NCCDs are highest for the two poorest quintiles, holding other characteristics constant. This indicates that high *potential* OOP expenditures might more strongly deter poor individuals from seeking treatment in the first place, since they have fewer financial means to pay for it. In addition, women are estimated to incur significantly higher OOP expenditures after correcting for selection. These results underline the importance of accounting for selection in estimating the determinants of OOP expenditures.

We do not find significant evidence of a selection effect in the second model (Table 6 columns 3–4) with relative OOPs as the dependent variable, although the *ρ* coefficient is negative as expected (*ρ* = –.08, p = .680). Controlling for other characteristics, wealth status does not significantly drive relative OOPs. No significant gender effect is visible, either.

## Discussion

This study investigated the heterogeneity across sex and wealth levels in health care utilization for treatment of non-communicable chronic diseases (NCCDs), the concomitant out-of-pocket expenditures, and distances travelled to health care facilities in rural Nigeria. Due to substantial heterogeneity in health care utilization, we employed a Heckman selection model to estimate NCCD-related expenditures taking into account self-selection in seeking formal health care.

6.2% of the population in our sample suffered from an NCCD. The most frequently reported NCCDs were musculoskeletal disorders and cardiovascular diseases. Precise estimates of the global prevalence of musculoskeletal disorders are difficult to obtain because standards in outcome definition and in data collection vary dramatically. In Sub-Saharan and North Africa, the point prevalence of low back pain, which is one of the most prevalent musculoskeletal conditions, was estimated to be 32%, with women being at higher risk to suffer from it [39]. A recent study evaluated the global burden of musculoskeletal disorders expressed as years lived with disability and showed that low back pain is the leading cause of years lived with disability at 10.7% [40]. The relatively high prevalence of self-reported cardiovascular diseases in this study mirrors the prevalence rates in the WHO Nigeria country profile [41]. Nevertheless, low disease awareness associated with low education and income can lead to substantial underreporting of prevalence [42,43]. A recent study in Kwara State, Nigeria, showed a 19% prevalence of hypertension among individuals 18 years and above, but only 8% of respondents with hypertension were aware of their condition [44]. Lack of access to health services sustains low levels of awareness and an absence of preventive care, thereby maintaining risk factor exposure and increasing the risk of end-stage disease [45].

Our results indicate that the poor are strained by a double burden of high out-of-pocket expenditures for treatment of NCCDs, combined with a lower rate of formal health care utilization. Longer distances to reach health care facilities prevent many, and the poor disproportionately, to seek formal care in the first place. While self-reported prevalence rates of NCCDs are highest among the poor, they have the lowest rate of health care utilization. Households in the poorest quintile incurred significant health expenditures in absolute terms, and expenditures relative to annual household consumption were 2.2 times higher for the poorest quintile than those of the wealthiest households. Using a Heckman regression model to account for selection into health care, we estimate significantly higher absolute NCCD-related expenditures for the lowest quintile compared to the three upper quintiles. This suggest that the high *potential* expenditures relative to income for the poorest constitute a major deterrent to seeking care especially for those least able to pay for it.

Naturally, our results depend on the correct specification of the Heckman selection model. A Strong identification of the Heckman model requires the presence of at least one instrumental variable explaining utilization of formal health care, which can be credibly excluded from the OOP equation. To achieve this, we used the distance to the nearest provider. This instrument is powerful as it explains negatively and significantly the utilization of formal health care (“participation”), as expected. After controlling for participation, we assume that this variable can reasonably be excluded from the decision on how much to spend on health expenditure: unlike distance to the chosen provider, this variable is unlikely to capture any unobserved characteristic of the quality of healthcare chosen that would explain the OOP expenditures. Identification relies on the assumption that the distance to the nearest provider affects the OOPs through participation in formal health care only, but has no direct effect on OOPs. This obviously only holds if travel costs are not included in OOPs, as is the case in our study.

Our results are in line with existing evidence showing that the poorest households in Nigeria carry the largest burden of health expenditures [46–48]. The limited financial affordability of health care for the poor is of great concern, especially in countries such as Nigeria where 54% of the population live in poverty [49] and formal financial risk protection against health expenditures is inaccessible to the majority of households. Health insurance coverage in Kwara state was only 0.8% at the time of the survey [44]. Private out-of-pocket expenditures accounted for over 70% of the estimated $10 per capita expenditure on health [50].

In terms of differences across sexes, women were 1.6-fold more likely to forego formal care than men when suffering from an NCCD. Women also incurred higher absolute and relative OOP expenditures, although differences across sexes were not statistically significant. These findings suggest that Nigerian women face a substantial gender bias in health care utilization. Further research is needed to fully understand how gender affects demand for health care related to NCCDs.

The risk of catastrophic health spending for NCCD-affected individuals was substantial: In 8.8% of NCCD cases, health expenditures exceeded 40% of their households’ capacity to pay. On average, 1.7% of the households in our full sample experienced catastrophic health spending due to the prevalence of NCCDs. This is not negligible: the only Sub-Saharan country with a higher rate reported by [30] in their cross-country study on catastrophic health spending was Zambia (2.3%). Among the chronically ill in our setting, vulnerability to financial catastrophe was particularly pronounced among the two poorest wealth quintiles at 10.8% for the lowest and 13.5% for the second quintile.

When seeking formal care, the poorest were most likely to go to a private provider. A potential explanation suggested by anecdotal evidence from Kwara and other Nigerian regions [46] is the possibility of paying in-kind or in installments at private providers but not in public hospitals. More respectful staff attitudes towards patients in private compared to public facilities may be another explanation [51]. Third, the choice for private providers may be partly explained by travel distance, since poor households lived on average slightly closer to a private than a public facility.

Transportation costs to visit a health provider represented about 8% of total health expenditures for NCCDs on average. However, the importance of transportation costs is not so much reflected in the observed amount that is spent, but rather in their “shadow price”. High transportation costs deter individuals from actually seeking care, in which case costs are not incurred. Geographical accessibility was linked to socio-economic status, such that the poorest lived furthest away from the nearest provider of either type. This represents an important barrier to health care, as our results show that a longer distance to the nearest health facility impedes households from seeking treatment.

Distances actually travelled did not significantly differ across sexes or quintiles. Respondents systematically bypassed the nearest clinic to seek treatment, irrespective of their gender or socio-economic status. This finding is in line with other studies on bypassing conducted amongst others in Tanzania [52] and Uganda [53]. Bypassing is generally related to poor quality of local health centers. The greater distance to good quality care potentially accounts for a substantial share of foregone formal care, particularly for those with fewer financial resources [54]. This calls for innovative solutions, in addition to quality improvement of local health centers. Among them, policy-makers have considered targeting isolated areas, subsidizing mobile health care units that unlock remote areas, or providing transport vouchers for free that cover travel costs to clinics. Improving geographical access may prove an essential strategy in the battle against NCCDs given the need for recurrent provider consultations that can drive up transportation costs substantially.

Taken together, the findings of this study emphasize the need for financial protection against health expenditures for the treatment of NCCDs, especially for the most vulnerable populations such as women and the poor. Low-cost health insurance may offer a potential solution, particularly if it provides coverage of preventive services and treatment of chronic disease. An increasing number of rigorous impact evaluations in developing countries shows that access to health insurance increases health care utilization and decreases health-related OOPs [55]. However, uptake of such schemes often remains low, even when heavily subsidized, particularly in the lowest wealth quintiles [56]. Further research is warranted to investigate how formal insurance schemes, in combination with quality upgrading, expansion of facilities to remote areas, or coverage of transportation costs, may enhance the demand for health insurance and health care services, and relieve part of the financial burden of chronic disease felt most heavily by women and the poor.

## Supporting Information

S1 File Minimal dataset for reproduction of results. Dataset containing variables used for analysis, in Stata format (version 13).
(ZIP)

S1 Table Types of self-reported NCCDs. Incidence of the different NCCDs in the sample: overall, by sex, and by asset-based wealth quintile. All numbers are row percentages, test statistics are based on Pearson’s χ^2^ test.
(DOCX)
