# Supplementary material for: The Financial Burden of Non-Communicable Chronic Diseases in Rural Nigeria: Wealth and Gender Heterogeneity in Health Care Utilization and Health Expenditures
Source: PLoS One. 2016 Nov 10;11(11):e0166121. doi: 10.1371/journal.pone.0166121 (PMC5104487; doi:10.1371/journal.pone.0166121)
Supplement: S1 Table — Incidence of the different NCCDs in the sample: overall, by sex, and by asset-based wealth quintile. All numbers are row percentages, test statistics are based on Pearson’s χ2 test. (DOCX) [file pone.0166121.s002.docx]

S1 Table. Type of self-reported NCCDs by sex and wealth quintile, in percent

|  | Cardiovascular Disorders | Musculoskeletal Disorders | Respiratory Disorders | Physical Disability | Diabetes | Allergy | Peptic Ulcer Disease | Others | **N** |
| --- | --- | --- | --- | --- | --- | --- | --- | --- | --- |
|  |  |  |  |  |  |  |  |  |  |
| **Total** | 13.7 | 46.4 | 7.0 | 9.5 | 3.6 | 4.8 | 5.0 | 10.1 | **358** |
|  |  |  |  |  |  |  |  |  |  |
| **Seks** |  |  |  |  |  |  |  |  |  |
| Male | 10.5 | 42.1 | 8.6 | 12.5 | 5.9 | 5.9 | 2.6 | 11.8 | **152** |
| Female | 16.0 | 49.5 | 5.8 | 7.3 | 1.9 | 3.9 | 6.8 | 8.7 | **206** |
|  |  |  |  |  |  |  |  |  |  |
| *χ² stat (df = 7)* | 14.13** |  |  |  |  |  |  |  |  |
|  |  |  |  |  |  |  |  |  |  |
| **Asset-based wealth quintile** |  |  |  |  |  |  |  |  |  |
| 1 (poorest) | 7.3 | 57.3 | 8.3 | 9.4 | 3.1 | 3.1 | 5.2 | 6.3 | **96** |
| 2 | 11.8 | 47.4 | 10.5 | 10.5 | 4.0 | 5.3 | 5.3 | 5.3 | **76** |
| 3 | 16.3 | 46.3 | 3.8 | 11.3 | 3.8 | 5.0 | 3.8 | 10.0 | **80** |
| 4 | 7.4 | 42.6 | 1.9 | 9.3 | 5.6 | 9.3 | 1.9 | 22.2 | **54** |
| 5 (richest) | 30.8 | 28.9 | 9.6 | 5.8 | 1.9 | 1.9 | 9.6 | 11.5 | **52** |
|  |  |  |  |  |  |  |  |  |  |
| *χ² stat.(df = 28)* | 27.98 |  |  |  |  |  |  |  |  |
|  |  |  |  |  |  |  |  |  |  |
| All numbers are row percentages. Significance values for Pearson’s χ² statistics: * p<0.1, ** p<0.05, *** p<0.01. ‘df’ is degrees of freedom | | | | | | | | | |
